# Supplementary material for: Industrial-scale prediction of cement clinker phases using machine learning
Source: Commun Eng. 2025 May 24;4:94. doi: 10.1038/s44172-025-00432-3 (PMC12103584; doi:10.1038/s44172-025-00432-3)
Supplement: Supplementary file 2 — Supplementary material [file 44172_2025_432_MOESM2_ESM.pdf]

# Supplementary Information to

# "Industrial-scale Prediction of Cement Clinker Phases using Machine Learning"

Sheikh Junaid Fayaz[1], Néstor Montiel-Bohórquez[2], Shashank Bishnoi[1], Matteo Romano[2], Manuele Gatti[2], N. M. Anoop Krishnan[1,3]

[1]*Indian Institute of Technology Delhi, Hauz Khas, 110016, New Delhi, India*
[2]*Politecnico di Milano, Department of Energy, Lambruschini 4A, 20156, Milan, Italy*
[3]*Corresponding authors: NMAK (krishnan@iitd.ac.in)*

## Supplementary A: Data

**Table S1:** Description of process parameters (PP) reported in DB1.The controllable PP are super-scripted with $*$

| Notation | Parameter | Description | Unit | Range |
|---|---|---|---|---|
| $P_1$ | Exit temp C1A | Gas outlet temperature (stage 1A cyclone) | °C | 0 - 600 |
| $P_2$ | Exit temp C1B | Gas outlet temperature (stage 1B cyclone) | °C | 0 - 600 |
| $P_3$ | Exit temp C2 | Gas outlet temperature (stage 2 cyclone ) | °C | 0 - 800 |
| $P_4$ | Exit temp C3 | Gas outlet temperature (stage 3 cyclone ) | °C | 0 - 900 |
| $P_5$ | Exit temp C4 | Gas outlet temperature (stage 4 cyclone ) | °C | 0 - 1000 |
| $P_6$ | Exit temp C5 | Gas outlet temperature (stage 5 cyclone ) | °C | 0 - 1200 |
| $P_7$ | PH gas outlet temp | Preheater gas outlet temperature | °C | 0 - 600 |
| $P_8$ | $O_2$ in raw gas at PH outlet | $O_2$ in raw gas measured at preheater outlet | % | 0 - 25 |
| $P_9$ | Temp of KF to PH | Raw mill outlet temperature | °C | 0 - 150 |
| $P^*_{10}$ | KF flow rate to PH | Total kiln feed includes fly ash and dust | tph | 0 - 550.0 |
| $P_{11}$ | Flue gas exit temperature | Calciner exit duct centered south temp | °C | 0 - 1370 |
| $P_{12}$ | Flue gas $O_2$ content at calciner outlet | Dry/wet basis not reported | % | 0 - 25 |
| $P_{13}$ | Solid's outlet temp at kiln inlet | Measured at rotary kiln inlet | °C | 700 - 1500 |
| $P^*_{14}$ | Calciner's fuel consumption | Calciner coal feeding | tph | 0 - 36 |
| $P_{15}$ | $O_2$ in the kiln gas at kiln inlet | Measured at rotary kiln inlet | % | 0 - 25 |
| $P_{16}$ | HM temp of lowest cyclone | Same as Stage 5 outlet temperature | °C | 0 - 1200 |
| $P^*_{17}$ | Total cooling air | Total cooling air | $Am^3h^{-1}$ | 0 - 630000 |
| $P^*_{18}$ | III Air temp at CC outlet | Heat exchanger inlet temperature #1 | °C | 0 - 650 |
| $P_{19}$ | Clinker production | Clinker flow rate (calculated) | tph | 0 - 100 |
| $P_{20}$ | Total flow of gas entering GCT | GCT wet air flow | CFM | 0 - 3000 |
| $P_{21}$ | Temp of gas entering GCT | GCT inlet temperature | °C | 0 - 500 |
| $P_{22}$ | Spray water used in GCT | GCT water flow | $m^3h^{-1}$ | 0 - 70 |
| $P_{23}$ | GCT outlet temp | GCT outlet temperature | °C | 0 - 500 |
| $P_{24}$ | Temp of gas entering the main fan | Measured at main fan inlet | °C | 0 - 600 |
| $P_{25}$ | Raw mill electric consumption | Raw mill motor power | kW | 0 - 6000 |
| $P_{26}$ | Pre-calciner outlet pressure | Preheater stage 5 pressure above kiln inlet | mbar | -50 - 5 |
| $P^*_{27}$ | Kiln burner primary air flow | I Air (including transport air) | $m^3h^{-1}$ | 0.0 – 14000.0 |
| $P^*_{28}$ | Calciner burner primary air flow | III Air flow rate | $m^3h^{-1}$ | 0.0 – 10000.0 |
| $P^*_{29}$ | Kiln coal feeding | Fuel consumption (kiln) | $MTh^{-1}$ | 0.0 – 20.0 |
| $P^*_{30}$ | Preheater ID fan speed | Rotational speed of each fan | % | 0.0 – 100.0 |
| $P^*_{31}$ | Rotary kiln drive power | Driver of rotary kiln | kW | 0.0 – 2340.0 |
| $P^*_{32}$ | Kiln inlet pressure | Kiln inlet pressure | mbar | -15 |
| $P_{33}$ | Raw mill fan inlet pressure at pt 1 | Raw mill inlet pressure 1 | mbar | -15 - 5 |
| $P_{34}$ | Raw mill fan inlet pressure at pt 2 | Raw mill inlet pressure 2 | mbar | -15 - 5 |

**Table S2:** Details of the KF, HM and clinker composition data reported in DB2

| Database | Composition | Unit | Min | Max | Mean | Standard deviation | Measurement technique | Measurement point |
|---|---|---|---|---|---|---|---|---|
| **Kiln feed** | CaO | wt. % | 38.17 | 44.30 | 42.43 | 0.36 | XRF | Before the preheater tower |
| | $SiO_2$ | wt. % | 10.36 | 15.04 | 13.02 | 0.26 | XRF | |
| | $Al_2O_3$ | wt. % | 2.38 | 4.01 | 2.96 | 0.11 | XRF | |
| | $Fe_2O_3$ | wt. % | 1.86 | 2.71 | 2.11 | 0.05 | XRF | |
| | MgO | wt. % | 1.31 | 2.71 | 2.08 | 0.19 | XRF | |
| | $SO_3$ | wt. % | 0.03 | 0.61 | 0.13 | 0.06 | XRF | |
| | $K_2O$ | wt. % | 0.31 | 0.55 | 0.43 | 0.03 | XRF | |
| | $Na_2O$ | wt. % | 0.00 | 0.18 | 0.06 | 0.02 | XRF | |
| | Cl | wt. % | -0.01 | 0.10 | 0.02 | 0.01 | XRF | |
| **Hot meal** | $SO_3$ | wt. % | -0.02 | 2.97 | 0.88 | 0.18 | XRF | Before entering the rotary kiln (calciner exit) |
| | $K_2O$ | wt. % | 0.38 | 4.00 | 1.51 | 0.22 | XRF | |
| | $Na_2O$ | wt. % | 0.07 | 11.73 | 0.17 | 0.11 | XRF | |
| | Cl | wt. % | 0.00 | 3.25 | 0.64 | 0.20 | XRF | |
| | Alite | wt. % | 0.00 | 62.75 | 2.22 | 1.48 | XRD | |
| | Belite | wt. % | 0.00 | 23.76 | 2.69 | 1.21 | XRD | |
| | Ferrite | wt. % | 0.00 | 20.45 | 1.31 | 0.77 | XRD | |
| **Clinker** | CaO | wt. % | 57.71 | 66.75 | 64.62 | 0.42 | XRF | Exit of clinker cooler outlet |
| | $SiO_2$ | wt. % | 18.66 | 27.10 | 20.89 | 0.29 | XRF | |
| | $Al_2O_3$ | wt. % | 4.57 | 7.33 | 5.24 | 0.17 | XRF | |
| | $Fe_2O_3$ | wt. % | 3.06 | 4.42 | 3.55 | 0.11 | XRF | |
| | MgO | wt. % | 1.90 | 4.17 | 3.25 | 0.36 | XRF | |
| | $SO_3$ | wt. % | 0.07 | 1.43 | 0.50 | 0.10 | XRF | |
| | $K_2O$ | wt. % | 0.41 | 0.87 | 0.60 | 0.06 | XRF | |
| | $Na_2O$ | wt. % | 0.00 | 0.39 | 0.08 | 0.04 | XRF | |
| | Cl | wt. % | -0.01 | 0.32 | 0.01 | 0.01 | XRF | |
| | Alite | wt. % | 0.00 | 78.33 | 60.03 | 3.68 | XRD | |
| | Belite | wt. % | 0.00 | 40.61 | 15.11 | 3.43 | XRD | |
| | Ferrite | wt. % | 0.00 | 42.23 | 14.31 | 1.03 | XRD | |

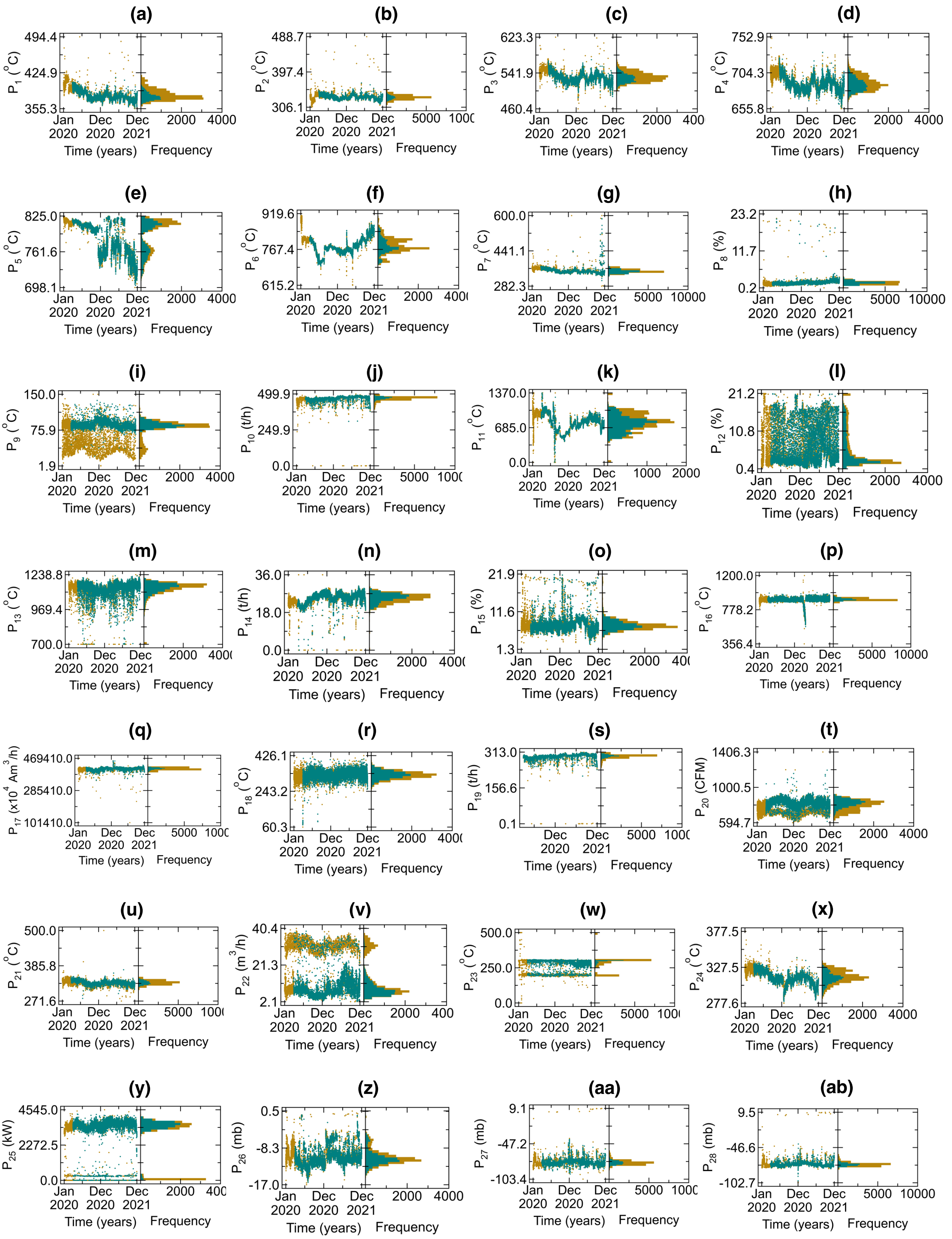


**Figure S1: Timeseries and distribution analysis of all process parameters used in the study**. Raw plant data is represented in yellow, while pre-processed data is shown in green. The notation for each process parameter on the y-axis corresponds to the labels provided in Table S1

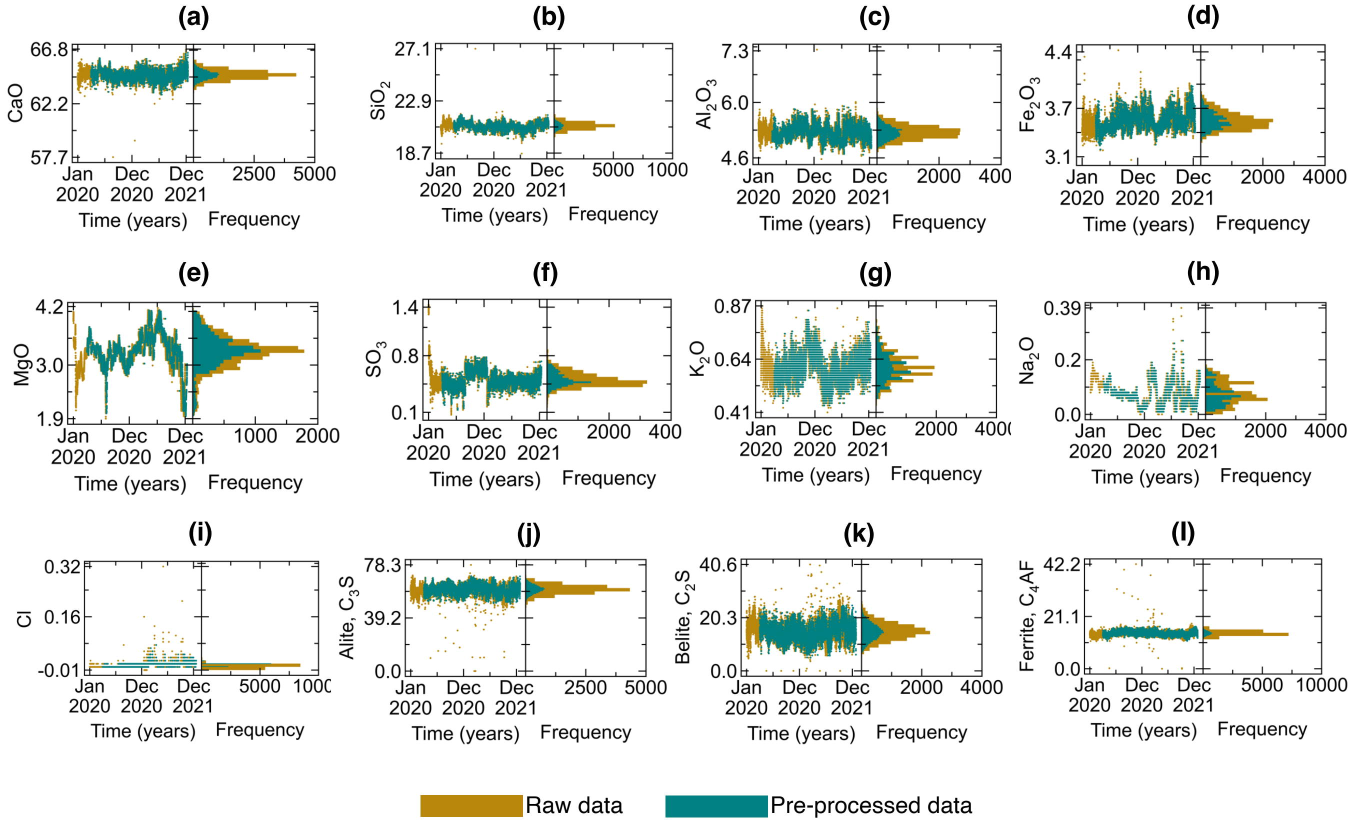


**Figure S2: Timeseries and distribution analysis of all compositions reported in the clinker database from the plant**. Raw plant data is represented in yellow, while pre-processed data is shown in green. The left and right subplots show scatter plots and histograms, respectively, for each of the reported clinker compositions.

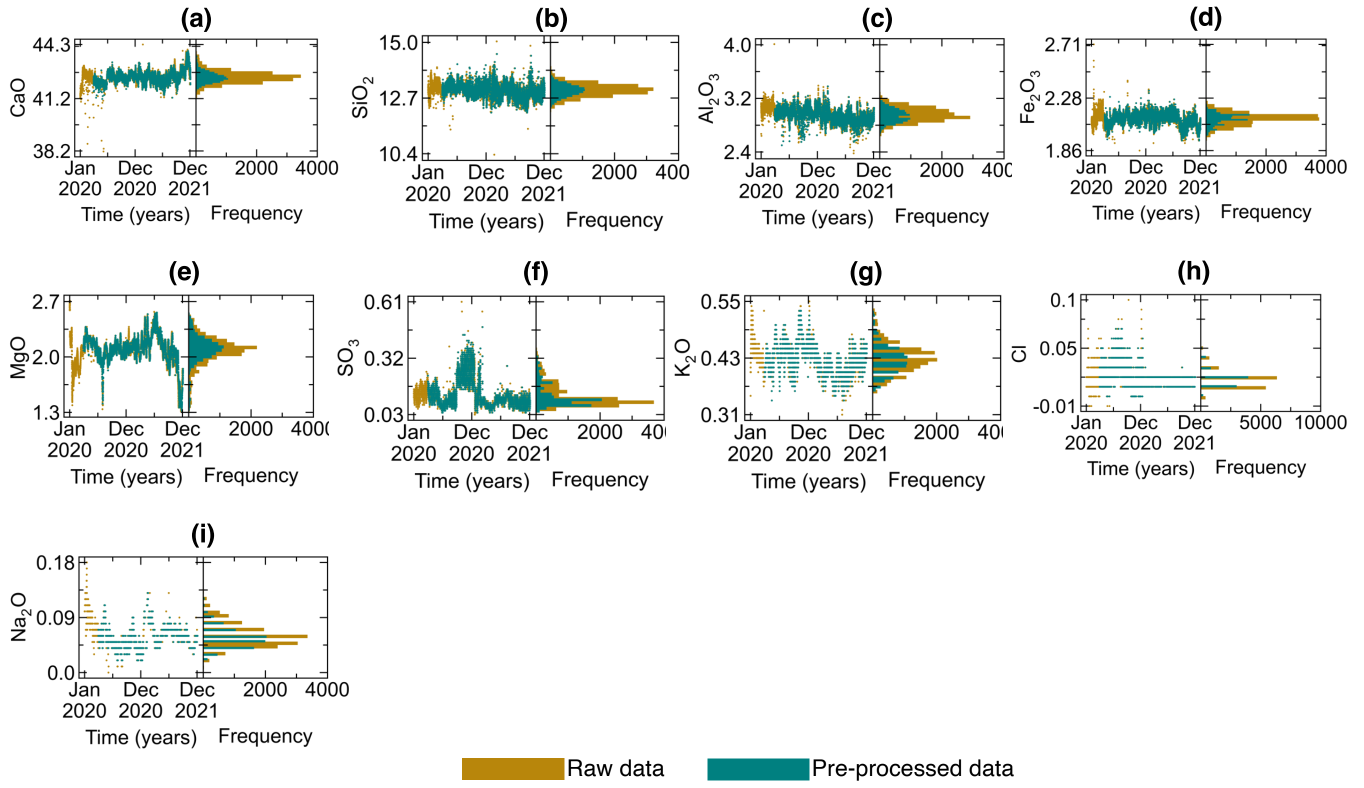


**Figure S3: Timeseries and distribution analysis of all compositions reported in the feed raw meal database from the plant**. Raw plant data is represented in yellow, while pre-processed data is shown in green. The left and right subplots show scatter plots and histograms, respectively, for each of the reported kiln feed compositions.

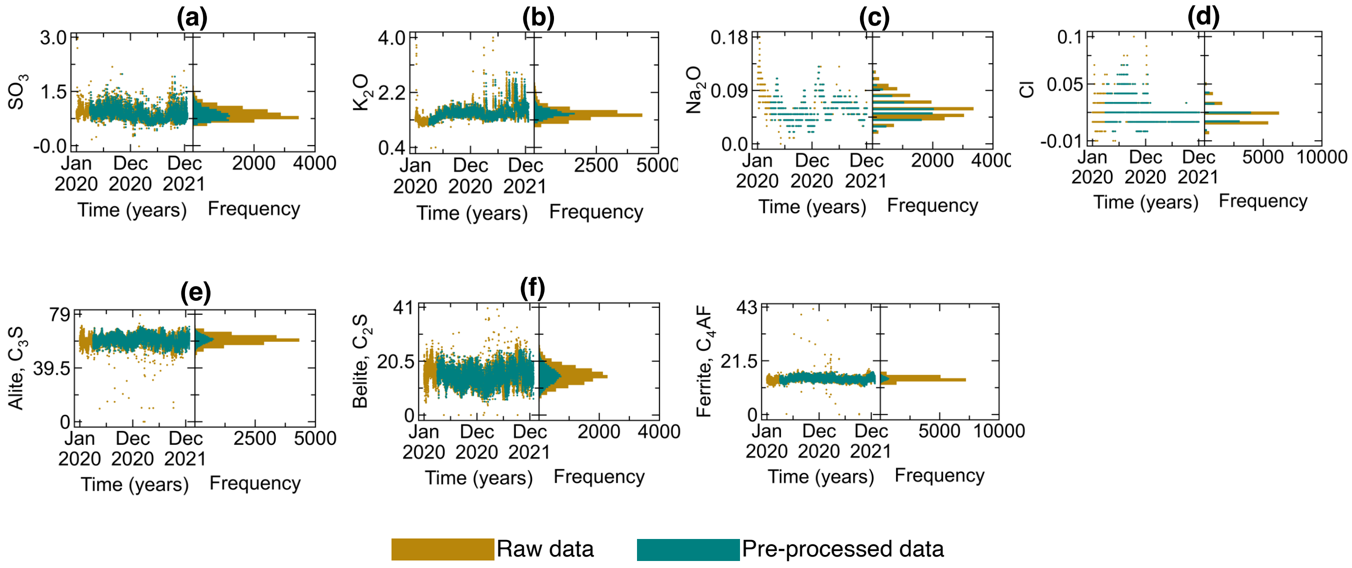


**Figure S4: Timeseries and distribution analysis of all compositions reported in the hot meal database from the plant**. Raw plant data is represented in yellow, while pre-processed data is shown in green. The left and right subplots show scatter plots and histograms, respectively, for each of the reported hot meal compositions.

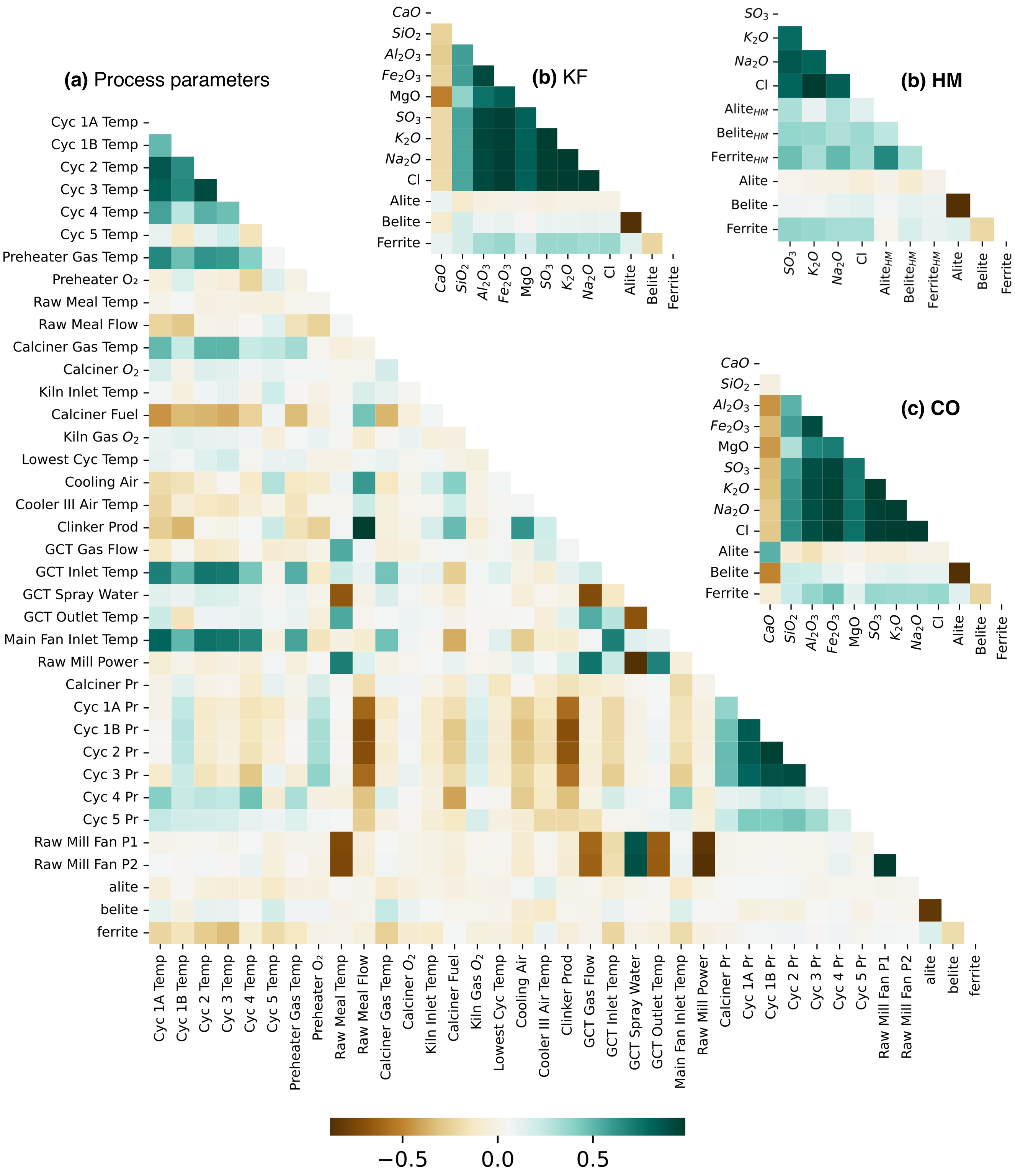


**Figure S5:** Heatmaps showing Pearson's correlation for (a) PP, (b) KF, (c) HM, (d) CO

# Supplementary B: ML algorithms

The ML algorithms were implemented for modeling the clinker mineralogy in this study are described in this appendix as following:

### Linear Regression (LR)

LR assumes a linear relationship between the predictors and the output. Thus making it unsuitable for application to nonlinear systems such as the operation of a cement kiln. For a data, $D_t$

$$D_t = \left\{ \underbrace{\begin{bmatrix} x_1^1 & x_2^1 & \dots & x_n^1 \\ x_1^2 & x_2^2 & \dots & x_n^2 \\ \vdots & \vdots & \ddots & \vdots \\ x_1^p & x_2^p & \dots & x_n^p \end{bmatrix}}_{\mathbf{X}}, \underbrace{\begin{bmatrix} y^1 \\ y^2 \\ \vdots \\ y^p \end{bmatrix}}_{\mathbf{Y}} \right\} \tag{S1}$$

the target variable can be represented using linear regression in the following form:[1]

$$Y = \theta_0 + \sum_{j=1}^{n} \theta_j X_j + \epsilon \tag{S2}$$

where $X_j = [x_j^1, x_j^2, \dots, x_j^i, \dots, x_j^p]^T$ , $\theta_0$ is the intercept, $\theta_j$ are the weights associated with each of the variable $X$ and $\epsilon$ is the uncertainty error. $\theta = [\theta_1, \theta_2, \dots, \theta_j, \dots, \theta_n]^T$ represents the model parameters which are determined by fitting a hyperplane to the data $D_t$ by minimizing the loss function which in this case is the residual sum of squares (RSS):[2]

$$\text{RSS}(\theta) = \sum_{i=1}^{p} [y^i - (\theta_0 + \sum_{j=1}^{n} \theta_j X_j)]^2 \tag{S3}$$

The predictor variables having more influence on the target output have higher weights and vice versa. However, in the linear regression method, weights for all input variables are non-zero, no matter how insignificant the contribution of the variable might be.

### Lasso regression

Overfitting[3] is a frequently encountered problem in ML that arises due to the large variance of the ML models when tested on unseen data. Such variance can be reduced by applying appropriate penalties in the loss function. Lasso, ridge, and elastic net regression algorithms are tweaked variants of linear regression algorithms wherein different penalizing measures are used in the loss functions to reduce model variance. In lasso regression, the weights of the input variables are penalized with a factor called the L1 norm, which shrinks the weight of insignificant variables to zero. The L1 norm is applied to the loss function (RSS) as follows:[4]

$$\hat{\theta}_{lasso} = \underset{\theta}{\text{argmin}} \left\{ \sum_{i=1}^{p} [y^i - (\theta_0 + \sum_{j=1}^{n} \theta_j X_j)^2] + \lambda_1 \sum_{j=1}^{n} |\theta_j| \right\} \tag{S4}$$

where $\lambda_1$ is a hyperparameter.

### Ridge regression

Ridge regression, like lasso, is a regularized version of LR. However, it uses the $L2$ norm on the squared weights as follows:

$$\hat{\theta}_{ridge} = \underset{\theta}{\text{argmin}} \left\{ \sum_{i=1}^{p} [y^i - (\theta_0 + \sum_{j=1}^{n} \theta_j X_j)^2] + \lambda_2 \sum_{j=1}^{n} |\theta_j|^2 \right\} \tag{S5}$$

where $\lambda_2$ is a hyperparameter. Note that the L2 norm reduces the weight of insignificant features but does not completely remove their contribution.

### Elastic net

The penalty-based regularization, i.e., lasso and ridge, can be further improved by increasing the number of penalty coefficients in the loss term. To this extent, Zou et al.[5] elastic net regression which combines the L1 norm and L2 norm as follows:

$$\hat{\theta}_{elastic} = \underset{\theta}{\text{argmin}} \left\{ \sum_{i=1}^{p} [y^i - (\theta_0 + \sum_{j=1}^{n} \theta_j X_j)^2] + \lambda_1 \sum_{j=1}^{n} |\theta_j| + \lambda_2 \sum_{j=1}^{n} |\theta_j|^2 \right\} \tag{S6}$$

Elastic net helps select the significant predictors by reducing the weights of insignificant predictors to 0.

**Random forest**

RF and XGBoost are a part of ensemble learning – a method that aggregates the results to form a multitude of decision trees. Bagging[6] and boosting[7] are the two most widely known methods for generating trees. Boosting involves the sequential growing of trees. The successive trees utilize the information generated from the preceding trees. At each decision tree, the dataset is modified to encode the information about the errors from the previously grown trees. The successive trees give extra weight to the points that are incorrectly predicted by the preceding trees. However, in the case of bagging, each tree is grown independently — they are grown simultaneously using different bootstrapped samples of the original dataset. For the training data, $D_t$ (Eqn S1 ), RF works as follows:[8] :

1. $N_{tree}$ bootstraped samples are drawn from the training data.
2. For each bootstraped sample, an unpruned decision tree is grown. Unlike a regular bagging scenario, where $M_{random} = n$, the RF algorithm randomly samples $M_{random}$ features, usually $\sqrt[2]{n}$ or $log_2(n)$, which are used to split each node. The best split among the sampled features is determined by information gain – gini index and entropy. 2. The final prediction( $\hat{\psi}_{RF}^{N_{tree}}(x)$) is made by averaging the predictions of all the decision trees as follows:

$$\hat{\psi}_{RF}^{N_{tree}}(x) = \frac{1}{N_{tree}} \sum_{i=1}^{N_{tree}} \psi_{RJ_i}(x) \tag{S7}$$

where $\hat{\psi}_{RF}^{N_{tree}}(x)$ denotes the averaged outcome of total $N_{tree}$ base learners and $\psi_{RF}(x)$ represents the prediction from individual trees.

The error rate of RF is calculated as follows:

1) At each bootstrap iteration, the data points not in the bootstraped sample, called the out-of-bag (OOB) data, are predicted by the tree grown with that bootstraped sample.
2) OOB predictions are aggregated to estimate the OOB error rate.

**XGBoost**

XGBoost is a boosting-based ensembling method wherein base learners (decision trees) are trained in succession to minimize the errors of the preceding trees. The training continues until a specified number of trees are grown or the objective function reaches an acceptable value. The growth of trees is governed by a greedy algorithm wherein a tree starts with 0 depth, and each tree node is split into leaves until a negative gain is reached. The following regularized objective function is minimized to learn the mapping:

$$\Gamma = \underbrace{\sum_i l(\hat{y}_i, y_i)}_{\textbf{Training loss}} + \underbrace{\sum_\kappa R(f_\kappa)}_{\textbf{Regularization penalty}} \tag{S8}$$

where $l(= \frac{1}{2p}(y_i - \hat{y}_i)^2)$ is the squared loss function, $\hat{y}_i$ and $y_i$ are the predicted and the actual values for the data $\{(x_i,\ y_i)\}_{i=1}^{p}$. $f_\kappa$ represents independent tree structures. In Eqn S8, the training loss focuses on improving accuracy, and the regularization penalty tries to trade off accuracy for generalizability. The regularization term penalizes the complexity of the tree as follows:

$$\underbrace{R(f_\kappa)}_{\textbf{Penalty for } k^{th} \textbf{ iteration}} = \underbrace{\alpha T}_{\text{Number of leaves}} + \underbrace{\frac{1}{2}\beta \sum_{j=1}^{T} w_j^2}_{\textbf{L2 norm of leaf scores}} \tag{S9}$$

T is the number of leaves in a tree. $\alpha$ and $\beta$ are the hyperparameters. Using Taylor's expansion, Eqn.S8 can be simplified to the following quadratic approximation for the $t^{th}$ iteration[9]

$$\tilde{\Gamma}^{(t)} = -\frac{1}{2} \sum_{j=1}^{T} \frac{Z_j^2}{\chi_j + \beta} + \alpha T \tag{S10}$$

where,

$$Z_j = \sum_{i \in I_j} \partial_{\hat{y}_i^{(t-1)}} l(y_i, \hat{y}_i^{(t-1)}) \tag{S11}$$

$$\chi_j = \sum_{i \in I_j} \partial^2_{\hat{y}_i^{(t-1)}} l(y_i, \hat{y}_i^{(t-1)}) \tag{S12}$$

So, fundamentally, XGBoost converts the optimization of a differential objective function to determine the minimum of a quadratic equation. Each base learner is fit to the training data by optimizing Eqn. S10. Moreover, the regularization term in the objective equips XGBoost to prevent overfitting. The $i^{th}$ data point at $t^{th}$ iteration is predicted by adding all the previous models as follows:

$$\underbrace{\hat{y}_i^{(t)}}_{\textbf{Final model (t)}} = \sum_{\kappa=1}^{t} f_\kappa(x_i) = \underbrace{\hat{y}_i^{(t-1)}}_{\textbf{Previous model (t-1)}} + \underbrace{f_t(x_i)}_{\textbf{New model being learnt (t)}} \tag{S13}$$

The final output of XGBoost ($\hat{y}$) is the summation of outputs from all the K models:

$$\hat{y} = \sum_{\kappa=1}^{K} f_\kappa(x), f_\kappa \in F(\text{Functional space of base learners}) \tag{S14}$$

**Support vector regression (SVR)**

SVR is an extension of the support vector machine (SVM) for regression. SVM determines a hyperplane which can linearly separate the data for classification. For data that is not linearly separable, SVM uses a mapping function, $\psi(x)$, to transform the data, $\{(x_i, y_i)\}_{i=1}^n$, into a higher dimension. The transformed data , $\{(\phi(x_i), y_i)\}_{i=1}^n$ , is linearly separable by the hyperplane ($f_{SVM}(x)$), expressed as:[10]

$$f_{SVM}(x) = \sum_{i=1}^{n} \omega_i^T \phi_i(x) + b \tag{S15}$$

where $\omega$ is the weight vector, and b is the offset. SVM uses kernel function ($K_{i,j}$) to compute the relation between points in higher dimensions as follows:

$$K_{i,j} = K(x_i, x_j) = \phi(x_i)^T \phi(x_j) \tag{S16}$$

In this study, $K(x_i, xj)$ was chosen to be radial basis function given as:

$$K_{i,j} = exp(-\gamma|x_i - x_j|^2) \tag{S17}$$

Unlike the aforementioned ML algorithms, which reduce the prediction error, SVM uses a soft margin approach, which reduces the acceptable margin in which the errors can lie. SVM minimizes the following constrained regularized objective function:

$$\Gamma_{SVM} = \min_{\omega,b,\xi} \left( \underbrace{\frac{1}{2}\omega^T\omega}_{\textbf{Error margin}} + \underbrace{\lambda \sum_{i=1}^{n} \xi_i}_{\textbf{Soft margin penalty}} \right) \quad \text{subject to} \quad \begin{cases} y_i - (\omega^T\phi(x_i) + b) \leq \epsilon + \xi_i, \\ (\omega^T\phi(x_i) + b) - y_i \leq \epsilon + \xi_i, \\ \xi_i \geq 0, \forall i \in \{1, ..., n\} \end{cases} \tag{S18}$$

$\lambda$ is a hyperparameter. Due to soft margin formulation, slack variable $\xi$ is introduced to measure the violation of error margin for miss-classified points. The first term of the objective function minimizes the error margin, and the penalty in the second term makes the model generalizable. Lower $\lambda$ gives less importance to classification mistakes, thereby making room for relaxing the error margin.

**Gaussian process regression (GPR)**

Gaussian process ($\mathcal{GP}$) is a collection of random variables (in this case, functions) such that any finite subset of the random variables has a joint Gaussian distribution. GPR is a non-parametric Bayesian ML algorithm wherein the input-output mapping function, $f : X \rightarrow Y$, is assumed to be distributed as a $\mathcal{GP}$. In GPR, the output $y$ of the function $f$ for input $x$ can be written as[11]

$$y = f(x) + \epsilon_{noise} \tag{S19}$$

The important assumptions are that the noise term, $\epsilon_{noise}$, follows a normal distribution with 0 mean and the function, $f(x)$, is distributed as a GP :

$$\epsilon_{noise} \sim \mathcal{N}\left(0, \sigma_{noise}^2\right) \quad \text{and} \quad f(x) \sim \mathcal{GP}\left(m(x), k(x, \acute{x})\right) \tag{S20}$$

A GP is completely defined as the mean function, $m(x)$, which is the mean of all the functions in the distribution, i.e., $m(x) = \mathbb{E}[f(x)]$, and the covariance function, $k(x, \acute{x})$, which computes the correlation between function outputs at different inputs $x$ and $\acute{x}$ :

$$k(x, \acute{x}) = \mathbb{E}\left[(f(x) - m(x))(f(\acute{x}) - m(\acute{x}))\right] \tag{S21}$$

In this study, the Radial basis function was chosen for computing the correlation as follows:

$$k_{rbf}(x, \acute{x}) = \sigma_f^2 exp\left(-\frac{||x - \acute{x}||^2}{2\lambda^2}\right) \tag{S22}$$

where $\lambda$ and $\sigma_f^2$ are hyperparameters. If $D = \{X_t, y_t\}$ is the training data, the outputs of the function, $f^* = [f_i^*|i = 1, ...n]^T$ for the new inputs $X^* = [x_i^*|i = 1, ...n]$, can be found from the joint normal distribution of previous observations, $y_t$ with the new function outputs, $f^*$ as follows:

$$\begin{bmatrix} y_t \\ f^* \end{bmatrix} \sim \mathcal{N}\left(0, \begin{bmatrix} K(X_t, X_t) + \sigma_{error}^2 I & K(X_t, X^*) \\ K(X^*, X_t) & K(X^*, X^*) \end{bmatrix}\right) \tag{S23}$$

where $K(X^*, X^*)$ is the covariance matrix between all the observed points,

$$K(X^*, X^*) = \begin{bmatrix} k(x_1^*, x_1^*) & k(x_1^*, x_2^*) & \dots & k(x_1^*, x_n^*) \\ (x_2^*, x_1^*) & k(x_2^*, x_2^*) & \dots & k(x_2^*, x_n^*) \\ \vdots & \vdots & \ddots & \vdots \\ (x_n^*, x_1^*) & k(x_n^*, x_2^*) & \dots & k(x_n^*, x_n^*) \end{bmatrix} \tag{S24}$$

and likewise for $K(X_t, X^*)$ and $K(X^*, X^*)$. Using conditional distribution corresponding to EqnS23, the predictive equations for GPR can be written as:[11]

$$f^*|X_t, y_t, X^* \sim \mathcal{N}\left( \underbrace{m(f^*)}_{\textbf{Predective mean}}, \underbrace{k(f^*)}_{\textbf{Predective covarience}} \right) \tag{S25}$$

$$\text{where} \quad m(f^*) = K(X^*, X_t)[K(X_t, X_t) + \sigma^2_{noise} I]^{-1} y_t \tag{S26}$$

$$\text{and} \quad k(f^*) = K(X^*, X^*) - K(X^*, X_t)[K(X_t, X_t) + \sigma^2_{noise} I]^{-1} K(X_t, X^*) \tag{S27}$$

**Neural network (NN)**

NN is a non-parametric mathematical model inspired by the biological functioning of neurons in the human brain. In this study, we employ a fully connected feedforward neural network consisting of an input layer, multiple hidden layers, and an output layer. The number of neurons in each layer is independent of each other. Each neuron has an activation function, $\mathbb{A}(\cdot) : \mathbb{R} \to \mathbb{R}$, which decides whether the neuron activates on signal reception. The neurons of the subsequent layers are connected via weighted links. The neurons of the input layer receive the input signal, and the output of the input layer is propagated to the neurons of hidden layers via the links. The output, $O_i$, of a neuron, $i$, in a hidden layer can be expressed as:[4]

$$O_i = \mathbb{A}\left( \sum_{j=1}^{N} \omega_{ij} x_j + T_i^{hid} \right) \tag{S28}$$

where $N$ is the number of neurons in the preceding $i-1$ layer, $\omega_{ij} x_j$ is the weighted sum of inputs to the neuron, $i$, from the neuron of preceding layer and $T_i^{hid}$ is the threshold of the hidden neuron $i$ . To account for the nonlinear input-output relation, a rectified linear unit (ReLU) activation function has been used in this study and is expressed as:

$$\mathbb{A}(\mathrm{X}) = \begin{cases} 0 & \text{for} X < 0, \\ X & \text{for} X \geq 0 \end{cases} \tag{S29}$$

# Supplementary C: Hyperparametric optimization and model training

The optimal values for the hyper parameters obtained after performing the 4 fold cross validation is shown in Table S3

The optimal values for the hyper parameters mentioned in Table S3 were obtained by performing four fold cross validation on the train data set using GridSearchCV library[12] in python. The optimized hyper parameter thus obtained were evaluated on the validation set before finally testing them on the unseen test set. Hyper parameters not mentioned in TableS3 were taken as per their default values. The comparison of the model performance on the train, val and test is shown in Fig S6 below.

We are showing the comparison of the ML models on the train and the test set using the parity plots as shown from FigS7 to FigS9.Also, the robustness of ML models was evaluated through 20 independent training iterations using different random seeds. Figure 2 in the main manuscript presents the mean predictions obtained from these 20 trained models. The gray bands enveloping the mean predictions in Figure 2 e,g,i represent the uncertainty in model predictions arising from variations across the 20 training runs. Performance of the best-performing models, along with error bars representing uncertainty, is shown in Fig. S10.

**Table S3: Hyperparametric optimization**.Optimized hyperparameters for ML models predicting alite, belite, and ferrite, obtained using GridSearchCV.

| **Model** | **Hyper parameters** | **Optimized value** | | |
|---|---|---|---|---|
| | | **Alite** | **Belite** | **Ferrite** |
| **Lasso** | alpha | 0.00001 | 0.0001 | 0.00001 |
| **Ridge** | alpha | 0.001 | 0.001 | 0.00001 |
| **Elastic net** | alpha | 0.00001 | 0.00001 | 0.00001 |
| | n_estimators | 300 | 300 | 700 |
| | random_state | 0 | 0 | 0 |
| | max_depth | 12 | 10 | 15 |
| | n_jobs | -1 | -1 | -1 |
| **Random forest** | ccp_alpha | 0.0001 | 0.0005 | 0.0001 |
| | max_features | 0.75 | 0.75 | 0.75 |
| | bootstrap | True | True | True |
| | min_samples_leaf | 4 | 6 | 3 |
| | min_samples_split | 8 | 10 | 8 |
| | random_state | 5 | 5 | 5 |
| | n_estimators | 500 | 450 | 600 |
| | learning_rate | 0.03 | 0.03 | 0.02 |
| | max_depth | 6 | 6 | 7 |
| **XGBoost** | min_child_weight | 10 | 11 | 8 |
| | subsample | 0.8 | 0.8 | 0.9 |
| | colsample_bytree | 0.8 | 0.8 | 0.9 |
| | reg_lambda | 7 | 8 | 5 |
| | reg_alpha | 3 | 3 | 1 |
| | n_jobs | -1 | -1 | -1 |
| | kernel | rbf | rbf | rbf |
| **SVR** | gamma | 0.001 | 0.001 | 0.0001 |
| | C | 100 | 100 | 1000 |
| | random_state | 3 | 3 | 3 |
| | normalize_y | False | False | False |
| | alpha | 1e-10 | 0.1 | 1e-10 |
| | n_restarts_optimizer | 0 | 10 | 0 |
| | kernel__k1 | RBF(length_scale=1) | $1^2$ | RBF(length_scale=1) |
| **GPR** | kernel__k2 | WhiteKernel(noise_level=1) | RBF(length_scale=10) | WhiteKernel(noise_level=1) |
| | kernel__k1__length_scale | 1 | 1 | 1 |
| | kernel__k1__length_scale_bounds | (1e-5, 1e5) | (0.01, 1000.0) | (1e-5, 1e5) |
| | kernel__k2__noise_level | 1 | 10 | 1 |
| | kernel__k2__noise_level_bounds | (1e-5, 1e5) | (0.001, 1000.0) | (1e-5, 1e5) |
| | kernel | RBF(length_scale=1) + WhiteKernel(noise_level=1) | 12* RBF(length_scale=10) | RBF(length_scale=1) + WhiteKernel(noise_level=1) |
| | epochs | 1000 | 1000 | 100 |
| | batch_size | 256 | 128 | 256 |
| | n_layers | 1 | 1 | 1 |
| | drop | True | True | True |
| **NN** | drate | 0.4 | 0.3 | 0.3 |
| | norm | false | false | false |
| | activation | ReLU | ReLU | LeakyReLu |
| | opt | Adam | Adam | SGD |
| | opt_params | lr : 5.815e-5 , weight decay: 4.811e-5 | lr:6.147e-5, weight_decay: 9.940e-5 | lr:4.459e-4, momentum: 1.193e-1 |
| | layers | 116 | 106 | 56 |

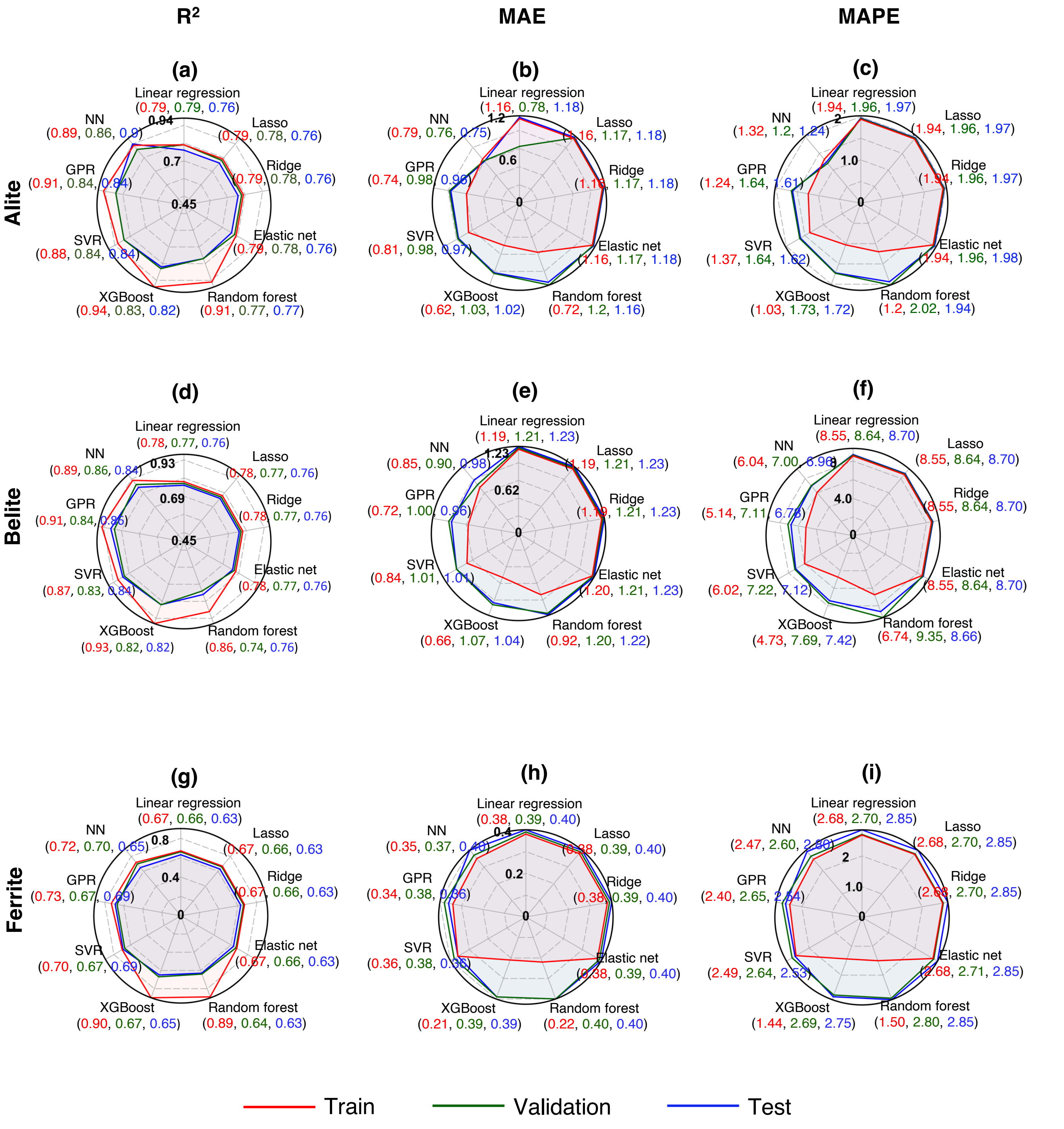


**Figure S6: Performance comparison of machine learning architectures on training (red), validation (green), and test (blue) sets**. a–c: Alite, with metrics for $R^2$, MAE, and MAPE, respectively. d-f: Belite, with metrics for $R^2$, MAE, and MAPE, respectively. g-i: Ferrite, with metrics for $R^2$, MAE, and MAPE, respectively.

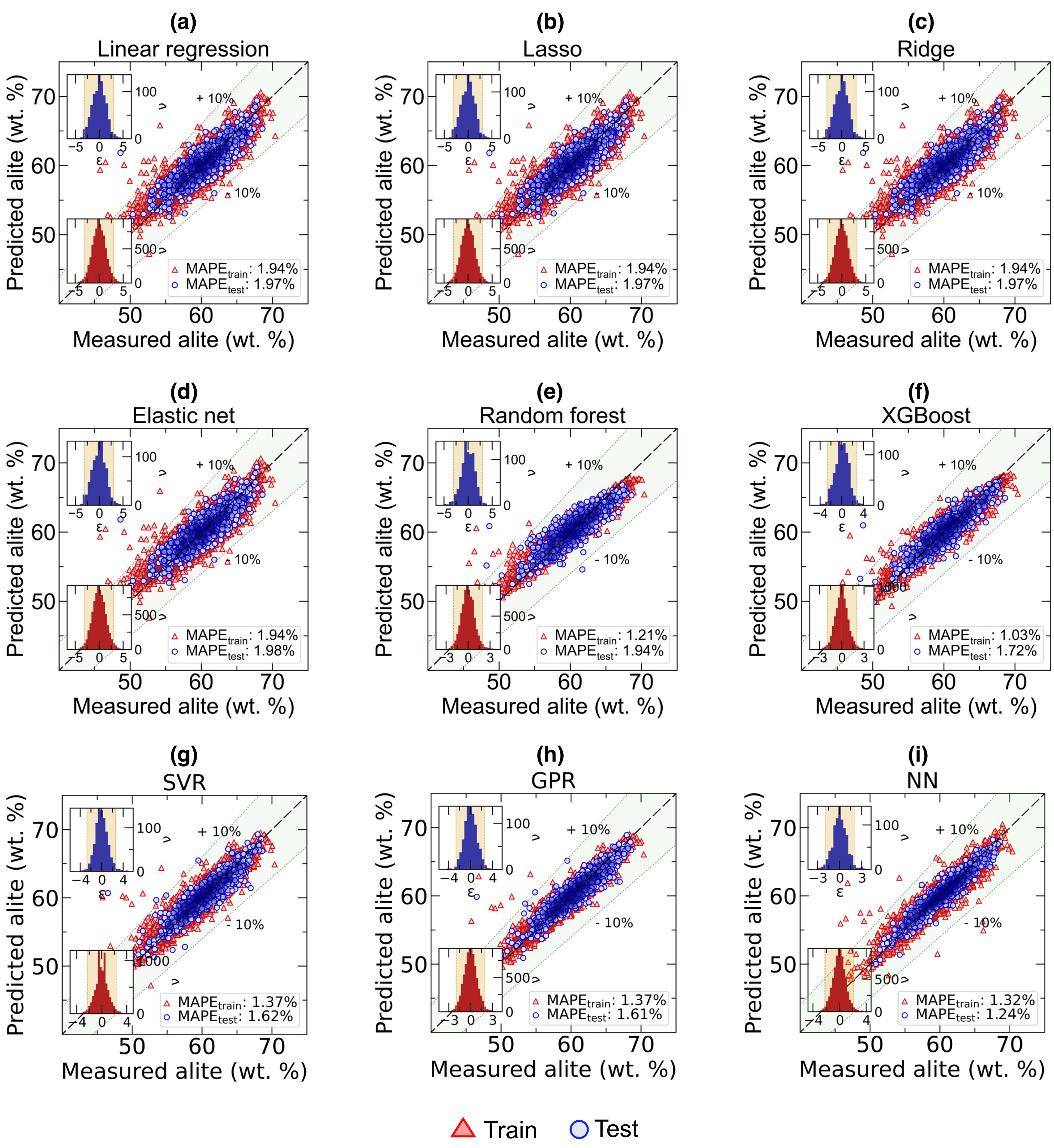


**Figure S7: Parity plots for comparing predicted versus measured clinker phases for different ML architectures**:**a** linear regression, **b** lasso, **c** ridge, **d** elastic net, **e** random forest, **f** XGBoost, **g** SVR, **h** GPR and **i** NN for predicting alite on the train (red) and the test set (blue).The dashed black black line represents the reference prediction line while the green region represents the 10% error margin from the reference.

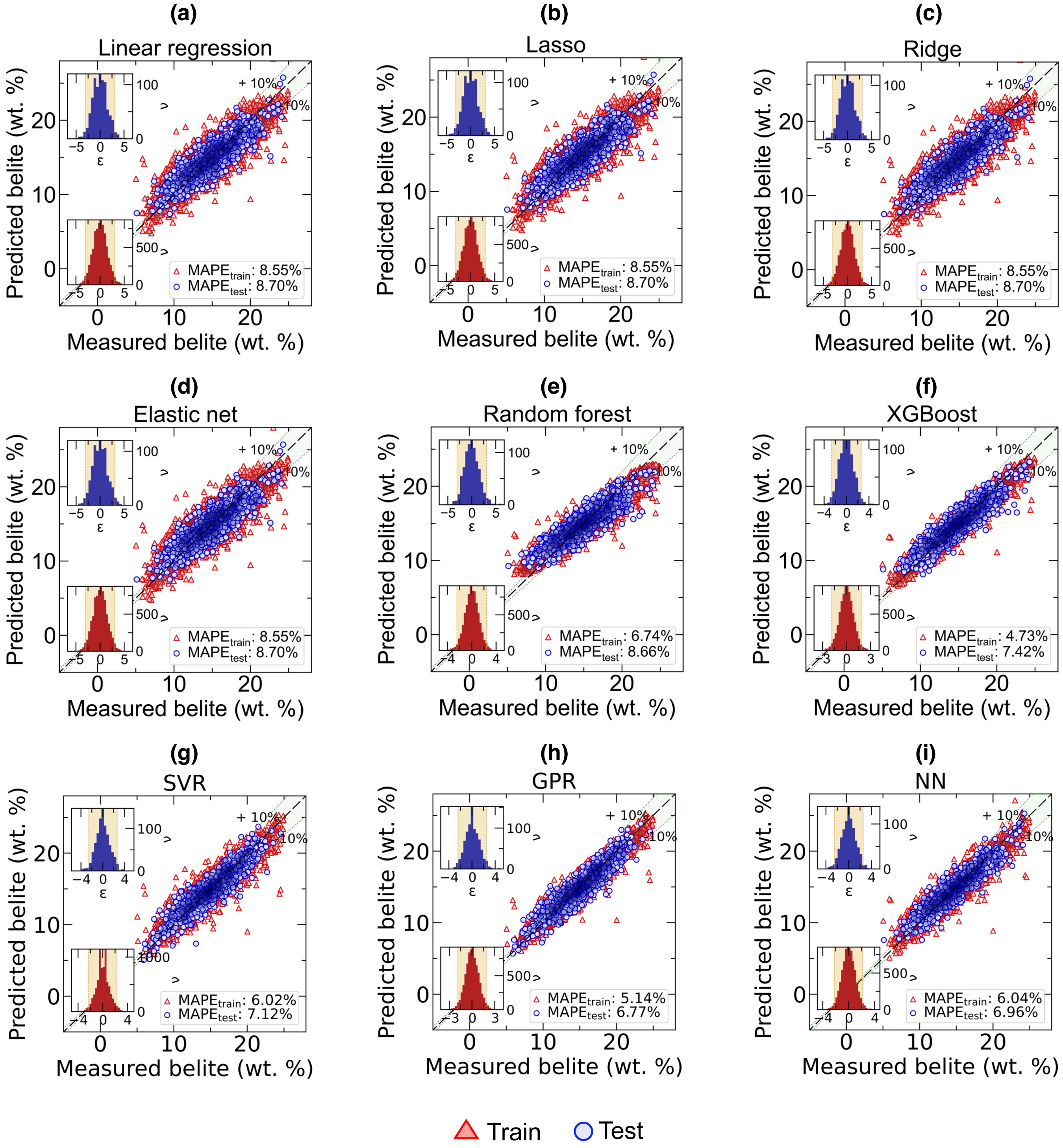


**Figure S8:** Performance of **(a)** linear regression, **(b)** lasso, **(c)** ridge, **(d)** elastic net, **(e)** random forest, **(f)** XGBoost, **(g)** SVR, **(h)** GPR and **(i)** NN for predicting belite on the train and the test set.

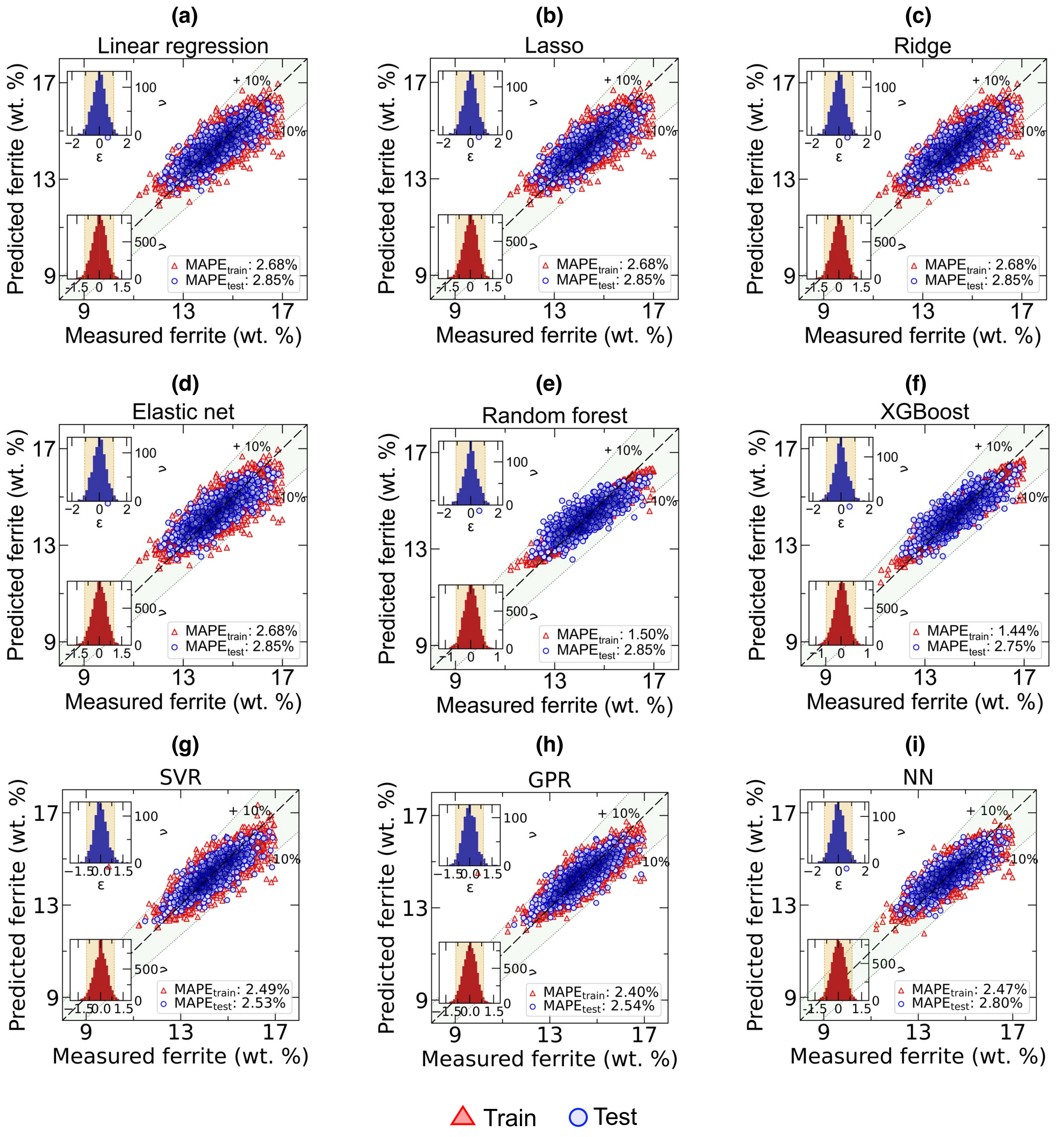


**Figure S9:** Performance of **(a)** linear regression, **(b)** lasso, **(c)** ridge, **(d)** elastic net, **(e)** random forest, **(f)** XGBoost, **(g)** SVR, **(h)** GPR and **(i)** NN for predicting ferrite on the train and the test set.

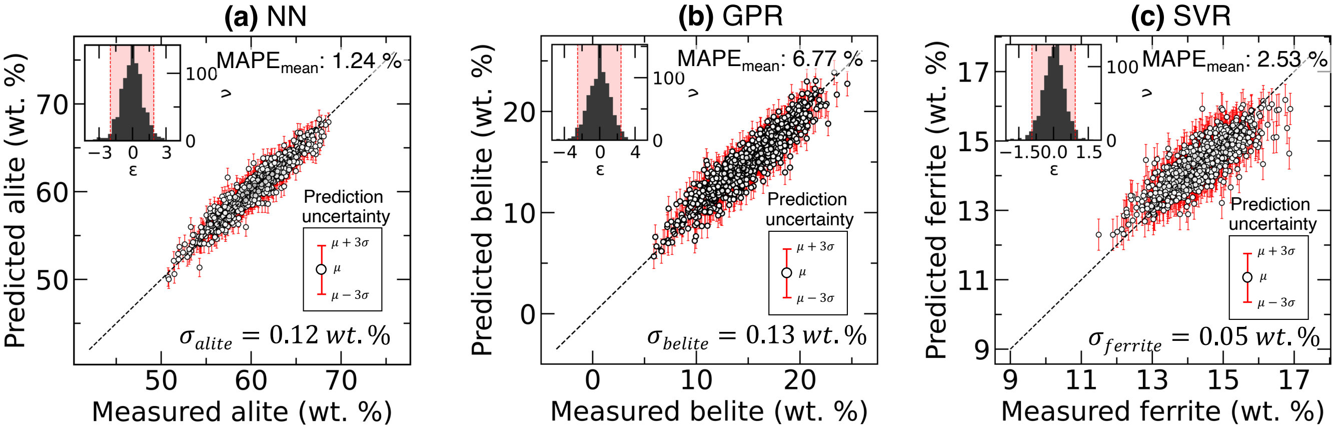


**Figure S10:** Performance of best performing models: (a) NN for alite, (b) GPR for belite, (c) SVR for ferrite) using all the 59 input features. Black circles represent the mean model prediction, while red error bars indicate model uncertainty ($\pm 3\sigma$)

# Supplementary D: Data-driven formulation of clinker equations

As shown in the main manuscript Figure 2, the Bogue-based predictions are significantly inconsistent with the measured XRD values. Due to oversimplified assumptions used in Bogue's approach,[13] the predictions have high uncertainty in most practical situations. Though the ML models outperform Bogue, they are not as straightforward and easy to apply. Where ML models need a computational machine or at least an interface, Bogue's prediction can be done with just pen and paper. This simplicity has kept Bogue's approach relevant so long from 1930$s$ despite the high uncertainty in its predictions. To this end, we present a data-driven approach to express the mineralogical composition of clinker as a linear algebraic equation. We used purely data-driven linear curve fitting to discover the equations from the data itself. The uncertainty in the phase estimates that is otherwise inherent to Bogue's approach due to the unrealistic assumptions is eliminated by learning the equations directly from the data. The general form of the equations formulated is as shown below:

$$\underbrace{\begin{bmatrix} y_1 \\ y_2 \\ y_3 \end{bmatrix}}_{\mathbf{Y}} = \underbrace{\begin{bmatrix} a_{11} & \cdots & a_{19} \\ \vdots & \ddots & \vdots \\ a_{31} & \cdots & a_{39} \end{bmatrix}}_{\mathbf{A}} \underbrace{\begin{bmatrix} x_1 \\ x_2 \\ x_3 \end{bmatrix}}_{\mathbf{X}} + \underbrace{\begin{bmatrix} b_1 \\ b_2 \\ b_3 \end{bmatrix}}_{\mathbf{B}} \tag{S30}$$

Where $[\mathbf{Y}]_{3\times1}$ is the clinker phase matrix, $[\mathbf{A}]_{3\times9}$ is the coefficient matrix, $[\mathbf{X}]_{3\times1}$ is the clinker oxides matrix, $[\mathbf{B}]_{3\times1}$ is the intercept matrix and $y_i$, $a_{ij}$, $x_j$, $b_i$ are the elements of the matrices as indicated in equation 1.
The clinker equations have been developed for the following cases below.

- **Case 1**: Includes only major CO
  In this case, the clinker phases are expressed as a linear algebraic combination of major clinker oxides. The equations developed are:

$$Alite = 2.97CaO - 4.5SiO_2 - 7.25SiO_2 + 0.05Fe_2O_3$$
$$Belite = -2.1CaO + 5.66SiO_2 + 6.15Al_2O_3 - 0.11Fe_2O_3$$
$$Ferrite = 0.02CaO - 0.28SiO_2 - 0.28Al_2O_3 + 3.82Fe_2O_3$$

  The equations can be represented in the matrix form as follows:

$$\begin{bmatrix} Alite \\ Belite \\ Ferrite \end{bmatrix} = \begin{bmatrix} 2.97 & -4.5 & -7.25 & 0.05 \\ -2.1 & 5.66 & 6.15 & -0.11 \\ 0.02 & -0.28 & -0.28 & 3.82 \end{bmatrix} \begin{bmatrix} CaO \\ SiO_2 \\ SiO_2 \\ Fe_2O_3 \end{bmatrix}$$

  In the subsequent cases, we gradually plug in the minor CO and the intercept into the matrix to determine how minor oxides and intercept contribute towards predicting the clinker phases.

- **Case 2**: Includes major CO and intercept.

$$\begin{bmatrix} Alite \\ Belite \\ Ferrite \end{bmatrix} = \begin{bmatrix} 4.84 & -3.62 & -4.47 & 1.52 \\ -4.71 & 4.5 & 2.39 & -1.89 \\ 0.65 & -0.3 & 0.52 & 4.24 \end{bmatrix} \begin{bmatrix} CaO \\ SiO_2 \\ SiO_2 \\ Fe_2O_3 \end{bmatrix} + \begin{bmatrix} -166.9 \\ -219.4 \\ -45 \end{bmatrix}$$

- **Case 3**: Includes only major CO and minor CO.

$$\begin{bmatrix} Alite \\ Belite \\ Ferrite \end{bmatrix} = \begin{bmatrix} 3.08 & -4.72 & -5.59 & 0.09 & -1.3 & 2.81 & -13.62 & 4.78 & -83.96 \\ -2.31 & 6.12 & 4.92 & -0.12 & -1.71 & -2.83 & 10.76 & -2.33 & 107 \\ 0.11 & -0.07 & 0.17 & 3.56 & -0.8 & 2.41 & -5.17 & -2.85 & -30 \end{bmatrix} \begin{bmatrix} CaO \\ SiO_2 \\ SiO_2 \\ Fe_2O_3 \\ MgO \\ SO_3 \\ K_2O \\ Na_2O \\ Cl \end{bmatrix}$$

- **Case 4**: Includes major CO, minor CO, and intercept.

$$\begin{bmatrix} Alite \\ Belite \\ Ferrite \end{bmatrix} = \begin{bmatrix} 4.72 & -3.15 & -4.28 & 3.08 & 0.33 & 2.4 & -7 & 5.96 & -75 \\ -5.17 & 4.04 & 1.7 & -3.48 & -0.94 & -5.11 & 3.76 & -4.3 & 86.46 \\ 0.05 & -0.13 & 0.1 & 3.41 & -0.9 & 2.29 & -5.35 & -2.99 & -27.37) \end{bmatrix} \begin{bmatrix} CaO \\ SiO_2 \\ SiO_2 \\ Fe_2O_3 \\ MgO \\ SO_3 \\ K_2O \\ Na_2O \\ Cl \end{bmatrix}$$

$$+ \begin{bmatrix} -166.9 \\ -219.4 \\ -45 \end{bmatrix}$$

The performance of the clinker equations formulated in case 1 to case 4 for predicting the clinker phases is shown in Figure 3. It can be seen that the MAPE for case 1 to case 4 does not vary significantly, which indicates that plugging in additional information into the case -1 equation in the form of intercept and minor CO does not contribute significantly towards improving the prediction accuracy. However, compared to the Bogue, the developed clinker equations have remarkably fewer prediction errors. As discussed in the main manuscript, Bogue tends to over-predict alite and under-predict belite and ferrite for the given plant. However, the developed clinker equations do not follow this specific bias. This is clear from the error histograms for the data-driven clinker equations in Figure 3 **(b)**, **(d)**and **(f)**, which are fairly symmetrical along the x-axis for all three phases.

Altogether, the motivation behind developing the clinker equations for the plant is to develop a middle ground between Bogue's equation-which are easy to fathom, easy to use, but high on error- and ML Models- which are not straightforward to use, infamous for working like a black box, but good at accuracy. The developed clinker equations preserve the simplicity (in terms of intelligibility and applicability) of Bogue while also making decent predictions. Though the predictive accuracy is not as remarkable as the ML models, it is still significantly better than the generic Bogue. Note that the clinker equations formulated here are plant-specific. Nonetheless, equations can be retrained for other plants also with minimal time and computation resources.

# Supplementary References


[1] G. James, D. Witten, T. Hastie, R. Tibshirani, An Introduction to Statistical Learning: with Applications in R, Springer Texts in Statistics, Springer US, New York, NY, 2021. doi:10.1007/978-1-0716-1418-1.
URL https://link.springer.com/10.1007/978-1-0716-1418-1

[2] C. M. Bishop, N. M. Nasrabadi, Pattern recognition and machine learning, Vol. 4, Springer, 2006.
URL https://link.springer.com/book/9780387310732

[3] B. Ghojogh, M. Crowley, The Theory Behind Overfitting, Cross Validation, Regularization, Bagging, and Boosting: Tutorial, arXiv:1905.12787 [cs, stat] (May 2023).
URL http://arxiv.org/abs/1905.12787

[4] N. Anoop Krishnan, S. Mangalathu, M. M. Smedskjaer, A. Tandia, H. Burton, M. Bauchy, Predicting the dissolution kinetics of silicate glasses using machine learning, Journal of Non-Crystalline Solids 487 (2018) 37–45. doi:10.1016/j.jnoncrysol.2018.02.023.
URL https://linkinghub.elsevier.com/retrieve/pii/S0022309318300905

[5] H. Zou, T. Hastie, Regularization and Variable Selection Via the Elastic Net, Journal of the Royal Statistical Society Series B: Statistical Methodology 67 (2) (2005) 301–320. doi:10.1111/j.1467-9868.2005.00503.x.
URL https://doi.org/10.1111/j.1467-9868.2005.00503.x

[6] L. Breiman, Bagging predictors, Machine Learning 24 (2) (1996) 123–140. doi:10.1007/BF00058655.
URL http://link.springer.com/10.1007/BF00058655

[7] P. Bartlett, Y. Freund, W. S. Lee, R. E. Schapire, Boosting the margin: A new explanation for the effectiveness of voting methods, The annals of statistics 26 (5) (1998) 1651–1686, publisher: Institute of Mathematical Statistics.
URL https://projecteuclid.org/journals/annals-of-statistics/volume-26/issue-5/Boosting-the-margin--a-new-explanation-for-the-effectiveness/10.1214/aos/1024691352.short

[8] A. Liaw, M. Wiener, Classification and Regression by randomForest 2 (2002).
[9] T. Chen, C. Guestrin, Xgboost: A scalable tree boosting system, in: Proceedings of the 22nd acm sigkdd international conference on knowledge discovery and data mining, 2016, pp. 785–794.
[10] C. Guardiani, E. Soranzo, W. Wu, Time-dependent reliability analysis of unsaturated slopes under rapid drawdown with intelligent surrogate models, Acta Geotechnica (2022) 1–26.
[11] C. E. Rasmussen, C. K. Williams, et al., Gaussian processes for machine learning, Vol. 1, Springer, 2006.
[12] sklearn.model_selection.GridSearchCV.
URL https://scikit-learn/stable/modules/generated/sklearn.model_selection.GridSearchCV.html
[13] T. I. Barry, F. P. Glasser, Calculations of Portland cement clinkering reactions, Advances in Cement Research 12 (1) (2000) 19–28, publisher: ICE Publishing. doi:10.1680/adcr.2000.12.1.19.
URL https://www.icevirtuallibrary.com/doi/full/10.1680/adcr.2000.12.1.19
